# Emergency Medical Services Support for Acute Ischemic Stroke Patients Receiving Thrombolysis at a Primary Stroke Center

**DOI:** 10.4137/jcnsd.s2221

**Published:** 2009-03-04

**Authors:** Byron R. Spencer, Omar M. Khan, Bentley J. Bobrow, Bart M. Demaerschalk

**Affiliations:** 1 Department of Neurology, Mayo Clinic; 2 Department of Internal Medicine, Mayo Clinic; 3 Department of Emergency Medicine, Mayo Clinic.

## Abstract

**Background:**

Emergency Medical Services (EMS) is a vital link in the overall chain of stroke survival. A Primary Stroke Center (PSC) relies heavily on the 9-1-1 response system along with the ability of EMS personnel to accurately diagnose acute stroke. Other critical elements include identifying time of symptom onset, providing pre-hospital care, selecting a destination PSC, and communicating estimated time of arrival (ETA).

**Purpose:**

Our purpose was to evaluate the EMS component of thrombolysed acute ischemic stroke patient care at our PSC.

**Methods:**

In a retrospective manner we retrieved electronic copies of the EMS incident reports for every thrombolysed ischemic stroke patient treated at our PSC from September 2001 to August 2005. The following data elements were extracted: location of victim, EMS agency, times of dispatch, scene, departure, emergency department (ED) arrival, recordings of time of stroke onset, blood pressure (BP), heart rate (HR), cardiac rhythm, blood glucose (BG), Glasgow Coma Scale (GCS), Cincinnati Stroke Scale (CSS) elements, emergency medical personnel field assessment, and transport decision making.

**Results:**

Eighty acute ischemic stroke patients received thrombolysis during the study interval. Eighty-one percent arrived by EMS. Two EMS agencies transported to our PSC. Mean dispatch-to-scene time was 6 min, on-scene time was 16 min, transport time was 10 min. Stroke onset time was recorded in 68%, BP, HR, and cardiac rhythm each in 100%, BG in 81%, GCS in 100%, CSS in 100%, and acute stroke diagnosis was made in 88%. Various diagnostic terms were employed: cerebrovascular accident in 40%, unilateral weakness or numbness in 20%, loss of consciousness in 16%, stroke in 8%, other stroke terms in 4%. In 87% of incident reports there was documentation of decision-making to transport to the nearest PSC in conjunction with pre-notification.

**Conclusion:**

The EMS component of thrombolysed acute ischemic stroke patients care at our PSC appeared to be very good overall. Diagnostic accuracy was excellent, field assessment, decision-making, and transport times were very good. There was still room for improvement in documentation of stroke onset and in employment of a common term for acute stroke.

## Background

Stroke is one of the top three killers in the United States and is one of the major causes of adult disability. Until 1996, and the Food and Drug Administration approval of intravenous tissue plasminogen activator (tPA), acute ischemic stroke was largely treated with supportive care. Now with treatment options such as intra-arterial thrombolysis, and mechanical clot retrieval, the era of rapid identification and appropriate transport of the stroke patient is here. This underscores the need for rapid evaluation by emergency medical personnel since they are the initial point of contact for 35%–70% of stroke victims.

Stroke patients that utilize 9-1-1 are more likely to arrive at hospital sooner, potentially giving the option for acute intervention. Often, other disorders can mimic stroke, and it is imperative that Emergency Medical Services (EMS) be able to recognize the most common ones such as seizure or hypoglycemia. With rapid identification, stabilization, and transport to a primary stroke center (PSC) with pre-notification, appropriate diagnostic studies and personnel can be mobilized sooner.

In 1998, the Consensus Development panel on stroke treatment of the National Institute of Neurological Disorders and Stroke developed a framework for improving stroke care, called the Stroke Chain of Recovery. This framework is made up of seven links in a chain for the treatment of stroke, and includes: detection, dispatch, delivery, door, data, decision, and drug. Based on this, EMS is an integral component in the overall sequence of events necessary for optimal stroke outcomes.

## Purpose

Our purpose was to evaluate the pre-hospital EMS component of thrombolysed acute ischemic stroke patient care at our PSC.

## Methods

In a retrospective manner we retrieved electronic copies of the EMS incident reports for every thrombolysed ischemic stroke patient treated at our PSC from September 2001 to August 2005. The following data elements were extracted: location of victim, EMS agency, on-scene time (from call to arrival at scene), transport time (time of departure from scene to arrival at PSC), recordings of time of stroke onset, pre-hospital blood pressure (BP), heart rate (HR), rhythm, blood glucose (BG), Glasgow Coma Scale (GCS), Cincinnati Stroke Scale (CSS) elements, Emergency Medical Technician (EMT) field diagnosis nomenclature, and transport decision making.

Scottsdale Fire Department (SFD) has 16 fire stations, 7 of which transport to Mayo Clinic Hospital (MCH) and Phoenix FD (PFD) has 63 fire stations, 6 of which routinely transport to MCH. Distances from fire stations to MCH vary from 6.0 miles (9 minutes) to 16.1 miles (21 minutes). All EMS in the region operate on a mutual aid system where they cross over geographical city boarders depending on the closest available unit. Fire Departments often respond together to calls on boarders of cities. PFD does their own patient transport and SFD does it through PMT Ambulance, a private ambulance provider. There is a computer aided dispatch system CAD which guides this from one central dispatch center in Phoenix. Each fire truck has a mobile computer terminal (MCT) with the location of the closest stroke center along with the center’s availability. The dispatch center also tracks each unit through the MCT by a global navigation system. We created a map of the Phoenix metropolitan region that included MCH and the geographic location of each of the subjects’ 9-1-1 calls.

The Mayo Clinic Institutional Review Board approved the use of the data collected as part of this minimal risk research study.

## Results

**Figure 1:**
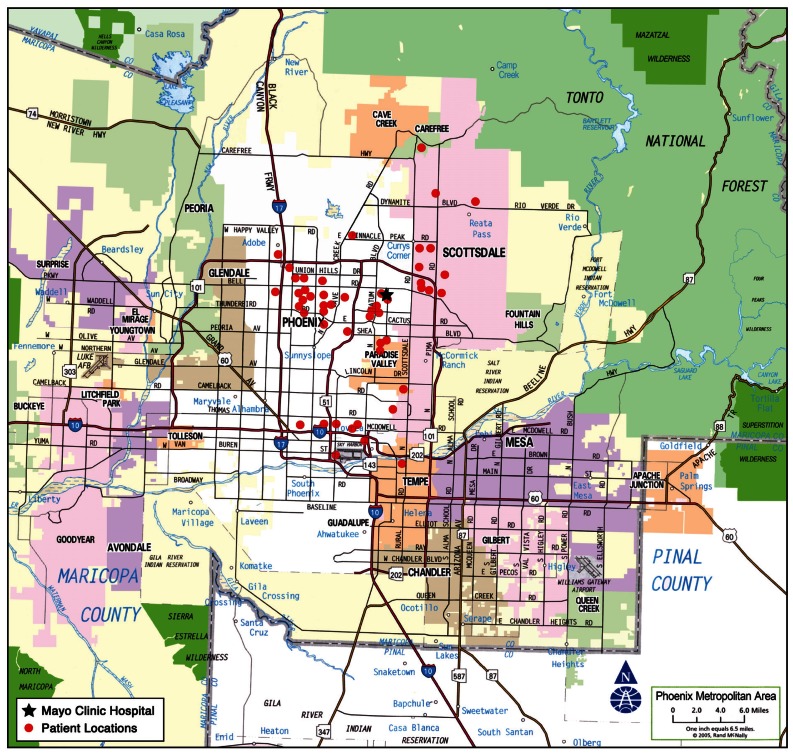


Eighty acute ischemic stroke patients received thrombolysis during the study interval. The mean age of the cohort was 69 years. Gender distribution was 68% female. The mean presenting NIHSS Score was 15. Sixty-five patients (65/80 = 81%) arrived by EMS. Two separate EMS agencies transported patients to our PSC. Mean dispatch-to-scene time was 6 min, on-scene time was 16 min, transport time was 10 min. Stroke onset time was recorded in 68%, BP, HR, and rhythm in 100%, BG in 81%, GCS in 100%, CPSS in 100%, and acute stroke diagnosis made in 88%. Various diagnostic terms were employed: Cerebrovascular Accident (CVA)/Possible CVA/Rule out CVA in 40%, Unilateral weakness/numbness in 20%, Loss of Consciousness (LOC)/Unresponsiveness in 16%, Stroke/Suspected Stroke in 8%, Other stroke terms in 4%. In 87% there was documentation of decision-making to transport to the nearest PSC and ETA. The Phoenix metropolitan region map displays the location of each 9-1-1 call in relationship to our PSC. The distances and travel times between local SFD and PFD fire stations and the Primary Stroke Center varies between 6 miles (9 minutes) and 16 miles (21 minutes) (See Fig. 1).

## Discussion

EMS is a vital link in the overall chain of stroke survival. The goal of this project was to evaluate the prehospital stroke care our EMS providers gave to acute ischemic stroke patients who received thrombolysis at our PSC.

Acute stroke is a dynamic process and fluctuation in clinical signs is a well-recognized entity, particularly within the first 24–48 hours.^1^ Accurate identification of stroke by prehospital personnel expedites triage of patients to acute stroke units and facilitate delivery of acute stroke therapies either in the hospital or in the community. Prehospital stroke recognition instruments were introduced in the mid 1990’s in the U.S.A. (Los Angeles Paramedic Stroke Scale [LAPSS] and Cincinnati Prehospital Stroke Scale Score [CPSS]).^2^–^5^ In our study prehospital personnel who included both EMT’s and paramedics used the Cincinnati Prehospital Stroke Scale to assess potential stroke victims. The CPSS appraises the presence or absence of speech abnormalities (assessing for dysarthria), facial palsy (checking for a symmetric smile) and asymmetric arm weakness (checking for unilateral arm drift) In conjunction with these signs and symptoms, noting the exact time of symptom onset was highly emphasized. At the time of the study, the recording of CPSS on incident reports was mandatory for Scottsdale Fire Department EMS, but not yet for Phoenix Fire Department EMS. Subsequently, it has become mandatory for both departments.

The evaluation of our data revealed that the incidence reports for all EMS transported ischemic stroke patients who received thrombolysis (65 subjects) contained all elements of the CPSS. This indicates that EMS personnel were diligent in using the various elements of the CPSS as a part of their standard field assessment to evaluate potential acute stroke patients.

The American Stroke Association produced an instructional video for EMS provider education that included information about the pathophysiology of stroke, the 3-hour timeframe for thrombolysis, the 7 links of the stroke chain of survival, the EMS protocol for recognition of acute stroke using the Cincinnati Prehospital Stroke Scale, recording of time of symptom onset (or the time the patient was last known to be symptom free), the assessment of serum glucose, and support of airway, breathing, and circulation. Additional video content included monitoring neurological status, obtaining intravenous access, using isotonic crystalloid solution, obtaining an electrocardiogram, treating seizures if applicable, prenotifying the treatment center of the estimated time of arrival, and providing rapid transport to the closest PSC emergency department. EMS providers were also shown a video enactment of an acute stroke victim and optimal prehospital treatment. Between December 1999 and January 2000, the EMS subcommittee designed a standardized stroke training module and slide set based on a stroke education needs assessment completed by 897 EMS personnel and 173 operators.^6^,^7^

Overall, our local EMS personnel did an excellent job at performing the critical actions needed in assessing and transporting a potential stroke patient. Some of the key data noted was that both the GCS and CPSS as well as BP, HR and rhythm, were assessed 100% of the time.

Our PSC receives approximately 320 EMS transported ischemic stroke patients annually. Overall, twelve percent receive intravenous tPA. Our PSC has consistently thrombolysed 100% of all tPA eligible stroke patients. The most common reasons for ineligibility are: time exceeding 3 hours, unknown time of onset, minor deficit, rapidly resolving deficit, anticoagulation and INR > 1.7. We reviewed the Arizona Stroke Prehospital Identification Registry and Education (ASPIRE) data which demonstrated how stroke onset to 9-1-1 call remained long, but 9-1-1 call to ED arrival was fast (average time from symptom onset to accessing 9-1-1 for those transported to a PSC was 113.1 minutes, average dispatch to arrival on scene was 6.4 minutes, average on-scene time was 16.6 minutes, and transport time was 11.1 minutes. Our study captured the selected EMS stroke assessment activity around our PSC, while the ASPIRE project captured the stroke assessment activity of multiple Arizona metropolitan FD EMS. Despite the difference in scope of the two studies, the times from 9-1-1 call to PSC arrival are quite similar.

The major time delay was onset of stroke symptoms to accessing 9-1-1.^8^,^9^ Focused public education on the signs and symptoms of acute stroke along with the role of the 9-1-1 emergency medical system for those having acute stroke symptoms is necessary.

Though overall our local EMS did an exceptional job at prehospital acute stroke care, there are areas upon which they could improve. A major emphasis in our EMS stroke training has been on the time urgent nature of stroke along with the need to identify as closely as possible the exact time of symptom onset. In evaluation of our data, only 68% of the time was time of symptom onset was recorded.

Through on-going stroke training with our EMS partners, this proportion can be improved. Of all the categories we assessed, this may be the most important, because it sets the initial tone for emergency department staff triaging potential interventional candidates.

Often the precise time of stroke symptom onset is difficult to elucidate but every attempt must be made to document this key information from patients, family, friends and bystanders. Though our data showed that close to 90% EMS transports provided prenotification to our PSC this number ideally should approach 100%. We are in the process of evaluating the entire Phoenix Metropolitan matrix of PSC’s to determine the percentage of stroke cases where our EMS partners document time onset.

Our EMS providers were trained to provide prenotification to our PSC for any suspected acute stroke patient presenting within 12 hours. This allows us to rapidly evaluate any potential candidate for acute intervention. Our center has initiated a stroke alert system that includes a series of events to decrease on scene to needle time (acute intervention including intravenous thrombolysis, intra arterial thrombolysis, or mechanical clot retrieval). The system begins when EMS personnel contacts the emergency department indicating a potential stroke patient is en route. From there emergency physicians prepare for rapid assessment of the patient, notify on call stroke neurologist, the CT scanner in radiology is readied, and laboratory personnel are notified so blood work can be expedited.

Field glucose assessment is performed to rule out hypoglycemia as a potential stroke mimic. Documentation of prehospital blood sugar assessment occurred in 81% of our patients and again can be improved with continued focus training.

Our study also looked at EMS run times. Tracking EMS times are important, because this allows us to analyze the various segments of our overall stroke chain of survival, and ensure no significant gaps exist in our system. In acute stroke, we must make sure that EMS personnel do not spend large blocks of time in evaluation, stabilization and transport, while a few minutes are not critical. The risk and benefits of responding to 9-1-1 calls and transporting patients with lights and sirens (code 3) must always be weighed. This is why the National Association of Emergency Dispatchers recommends an urgent response EMS stroke response to suspected stroke calls and to transport without lights and sirens.

Finally, the EMS terms used to describe suspected stroke patients were varied, ranging from “suspected stroke” to “unilateral weakness”. For clarification, our on-going EMS training stresses that prehospital personnel utilize the common term *stroke* as often as possible.

Limitations of this study include the retrospective nature of the evaluation, the narrow spectrum of stroke patients studied (no cerebral hemorrhage patients, no cerebral ischemia patients who did not receive thrombolysis, and no stroke mimic patients), the small sample size, and the inability to truly assess the utility of EMS evaluations (true positive, false positive, true negative, false negative, sensitivity, specificity, likelihood ratio).

## Conclusion

EMS delivered prehospital acute stroke care for the suspected acute ischemic stroke patient recipients of thrombolysis at our PSC is uniformly outstanding. The results of this survey show that overall EMS providers have a satisfactory understanding of acute stroke care. Diagnostic accuracy is excellent, field assessment; care, decision-making, and transport times are very good. There continues to be room for improvement in documentation of stroke onset, BG, and in employment of the common term *stroke* for suspected acute stroke patients.

## References

[1] Mohd Nor A, McAllister C, Louw SJ, Dyker AG, Davis M, Jenkinson D, Ford GA (2004). Agreement Between Ambulance Paramedic—and Physician— Recorded Neurological Signs With Face Arm Speech Test (FAST) in Acute Stroke Patients.

[2] Kothari RU, Pancioli A, Liu T, Brott T, Broderick J (1999). Cincinnati Prehospital Stroke Scale: reproducibility and validity. Ann Emerg Med.

[3] Kidwell CS, Saver JL, Schubert GB, Eckstein M, Starkman S (1998). Design and retrospective analysis of the Los Angeles Prehospital Stroke Screen (LAPSS). Prehosp Emerg Care.

[4] Kidwell CS, Starkman S, Eckstein M, Weems K, Saver J (2000). Identifying stroke in the field: prospective validation of the Los Angeles Prehospital Stroke Screen (LAPSS). Stroke.

[5] Harbison J, Hossain O, Jenkinson D, Davis J, Louw SJ, Ford GA (2003). Diagnostic accuracy of stroke referrals from primary care, emergency room physicians, and ambulance staff using the face arm speech test. Stroke.

[6] Bobrow BJ, Demaerschalk BM, Wood JP, Montgomery C, Clark L (2007). Assessment of emergency medical technicians serving the Phoenix metropolitan matrix of primary stroke centers. Stroke.

[7] Demaerschalk BM, Bobrow BJ, Paulsen M, Phoenix Operation Stroke Executive Committee (2008). Development of a metropolitan matrix of primary stroke centers: the Phoenix experience. Stroke.

[8] Bobrow B, Demaerschalk B, Shimmin SR, Clark L, McKinzie G (2007). Utilization of a prehospital stroke scale in an urban matrix of primary stroke centers. Prehosp Emerg Care.

[9] Bobrow BJ, Demaerschalk BM, Shimmin SR, Clark L, Aguilar MI, Ingall TJ (2007). Prehospital time intervals in a metropolitan matrix of primary stroke centers: Where has the time gone?. Stroke.

